# Supplementary material for: Content-rich biological network constructed by mining PubMed abstracts
Source: BMC Bioinformatics. 2004 Oct 8;5:147. doi: 10.1186/1471-2105-5-147 (PMC528731; doi:10.1186/1471-2105-5-147)
Supplement: Additional File 2 — The original results of the above study (non-essential files are deleted to keep the file size under the limit set by BMC bioinformatics). [file 1471-2105-5-147-S2.bz2 › chilibotAdditionalFile2/dip40/463ID11259614E10490/html/CXCL11_CXCR3.html]

 


 **CXCL11** and **CXCR3** 
  
Found 66 abstracts in PubMed, retrieved 40.  
 

 What does Google say? 
 PDF only 
| .edu only 

---

**Interactive relationship** (e.g. stimulation, inhibition, etc)

**Inhibitory relationship**- Moreover, murine  **CXCL11**  blocked the chemotactic effect of human  **CXCL11**  on human  **CXCR3**  transfectants.  Ref: 11500837 Eur J Immunol, 2001
- Processing of IP 10  [ **CXCR3** ]  and I TAC  [ **CXCL11** ]  by CD26 DPP IV resulted in reduced  **CXCR3**  binding properties, loss of calcium signaling capacity through  **CXCR3** , and more than 10 fold reduced chemotactic potency.  Ref: 11739156 Blood, 2001
- The biological activity of this truncated form of  **CXCL11**  was greatly reduced, both in calcium mobilization using  **CXCR3**  expressing CHO cells as well as its chemotactic activity for  **CXCR3**  expressing T cells.  Ref: 11559369 Eur J Biochem, 2001
- Truncated  **CXCL11**   **CXCL11**  3 73 had an eightfold reduced potential to bind and to regulate  **CXCR3** , but was completely inactive in calcium flux and chemotaxis assays.  Ref: 12101279 J Leukoc Biol, 2002
- An Alternatively Spliced Variant of  **CXCR3**  Mediates the Inhibition of Endothelial Cell Growth Induced by IP 10  [ **CXCR3** ] , Mig, and I TAC  [ **CXCL11** ] , and Acts as Functional Receptor for Platelet Factor 4.  Ref: 12782716 J Exp Med, 2003
- We alsodemonstrate that the  **CXCR3**  ligands inhibit CCR3 mediated functional responses of both human eosinophils and CCR3 transfectants induced by all three eotaxins, with  **CXCL11**  being the most efficacious antagonist.  Ref: 12884299 Eur J Immunol, 2003
- I TAC  [ **CXCL11** ]  4 73, which lacks the first three residues, has no agonistic activity but competes for the binding of I TAC  [ **CXCL11** ]  to  **CXCR3**  bearing cells and inhibits migration and Ca 2 changes in such cells in response to stimulation with I TAC  [ **CXCL11** ] , IP10  [ **CXCR3** ] , and Mig.  Ref: 12417585 J Biol Chem, 2003
**Neutral relationship**- Our data demonstrate that I TAC  [ **CXCL11** ]  is the physiological inducer of  **CXCR3**  internalization and suggest that chemokine receptor internalization occurs in physiological settings, such as leukocyte contact with an activated endothelium.  Ref: 11739530 J Immunol, 2001
- The CXC chemokines monokine induced by IFN gamma MIG CXC chemokine ligand CXCL 9, IP 10  [ **CXCR3** ]  CXCL10, and IFN inducible T cell alpha chemoattractant ITAC  **CXCL11**  act through their shared receptor,  **CXCR3** .  Ref: 12097412 J Immunol, 2002
- A potent antagonist for  **CXCR3**  was obtained by NH 2 terminal truncation of I TAC  [ **CXCL11** ] .  Ref: 12417585 J Biol Chem, 2003
- This correlated with studies using recombinant chemokines, which revealed that I TAC  [ **CXCL11** ]  was the most potent inducer of  **CXCR3**  down regulation and of transendothelial migration.  Ref: 11739530 J Immunol, 2001
- Here, the biological activity and expression patterns of murine  **CXCL11**  relative to CXCL9 MIG and CXCL10 IP 10  [ **CXCR3** ]  crg 2, the other two  **CXCR3**  ligands, were assessed.  Ref: 11500837 Eur J Immunol, 2001
- We have explored the structure function relationship of the  **CXCR3**  ligands, in particular of I TAC  [ **CXCL11** ] , which has highest affinity for  **CXCR3**  and is the most potent agonist.  Ref: 12417585 J Biol Chem, 2003
- We therefore characterized constitutive and regulated epithelial expression of the CXC chemokines IP 10  [ **CXCR3** ] , I TAC  [ **CXCL11** ]  and Mig, which specifically attract  **CXCR3**  expressing CD4 T cells.  Ref: 11737065 Clin Exp Immunol, 2001
- Human microvascular endothelial cell line 1 HMEC 1, transfected with either the known  **CXCR3**  renamed  **CXCR3**  A or  **CXCR3**  B, bound CXCL9, CXCL10, and  **CXCL11** .  Ref: 12782716 J Exp Med, 2003
- Distinct T cell subsets express different chemokine receptors, with type 2 cells mostly attracted by eotaxin, MDC, TARC and I 309, and type 1 cells sensitive to IP 10  [ **CXCR3** ] , Mig, I TAC  [ **CXCL11** ] , RANTES and MIP 1beta.  Ref: 11876523 Arch Dermatol Res, 2002
- The interferon IFN inducible chemokines, specifically, IFN gamma inducible protein 10 IP 10  [ **CXCR3** ] , monokine induced by IFN gamma Mig, and IFN inducible T cell alpha chemoattractant I TAC  [ **CXCL11** ] , share a unique CXC chemokine receptor  **CXCR3** .  Ref: 11739156 Blood, 2001
- Hybrid chemokines between I TAC  [ **CXCL11** ]  and IP10  [ **CXCR3** ]  were used to identify regions responsible for the higher activity of I TAC  [ **CXCL11** ] .  Ref: 12417585 J Biol Chem, 2003
- **CXCR3** , known to have four ligands IFN gamma inducible protein 10 gamma IP 10  [ **CXCR3** ] , monokine induced by IFN gamma Mig, I TAC  [ **CXCL11** ] , and 6Ckine, is predominantly expressed on memory activated T lymphocytes.  Ref: 11591765 J Immunol, 2001
- The chemokine receptor  **CXCR3**  is predominantly expressed on T lymphocytes, and its agonists CXCL9, CXCL10 and  **CXCL11**  are IFN gamma inducible chemokines that promote Th1 responses.  Ref: 12884299 Eur J Immunol, 2003
- Furthermore, no cell surface expression of  **CXCR3**  was detectable by flow cytometry, and the binding of 125I labeled IP 10  [ **CXCR3** ]  CXCL10 to these cells was not competed by the other high affinity ligands for  **CXCR3** , monokine induced by IFN gamma CXCL9, and I TAC  [ **CXCL11** ]   **CXCL11** .  Ref: 11714827 J Immunol, 2001
- Previous studies have shown that the CXC chemokine, IFN gamma inducible T cell alpha chemoattractant I TAC  [ **CXCL11** ] , was chemotactic for IL 2 activated human T lymphocytes, which express abundant  **CXCR3** .  Ref: 12055261 J Immunol, 2002
- Murine  **CXCL11**  also evoked calcium mobilization in CHO cells transfected with human  **CXCR3**  and was chemotactic for  **CXCR3**  expressing human T lymphocytes as well as for 300 19 pre B cells transfected with human or murine  **CXCR3** .  Ref: 11500837 Eur J Immunol, 2001
- Interactions between IP 10  [ **CXCR3** ]  CXCL10, Mig CXCL9 and I TAC  [ **CXCL11** ]   **CXCL11**  and their shared receptor,  **CXCR3** , seem to be responsible not only for Th1 cell infiltration in acute allograft rejection and in proliferative GN, but also for mesangial cell proliferation typical of the latter condition.  Ref: 12508169 G Ital Nefrol< MedlineTA>G Ital Nefrol, 2001
- I TAC  [ **CXCL11** ] , IP10  [ **CXCR3** ] , and Mig are interferon gamma inducible CXC chemokines that share the same G protein coupled receptor  **CXCR3** , which is preferentially expressed on Th1 lymphocytes.  Ref: 12417585 J Biol Chem, 2003
- Murine interferon inducible T cell alpha chemokine I TAC  [ **CXCL11** ]  is a potent non ELR Cys X Cys CXC chemokine that predominantly attracts activated T lymphocytes and binds to the receptor  **CXCR3** .  Ref: 11896933 Scand J Immunol, 2002
- Calcium mobilization and chemotaxis experiments demonstrated that murine  **CXCL11**  stimulated murine  **CXCR3**  at much lower doses than murine CXCL9 or murine CXCL10.  Ref: 11500837 Eur J Immunol, 2001
- Translational studies in a murine model demonstrated increased expression of MIG CXCL9, IP 10  [ **CXCR3** ]  CXCL10, and ITAC  **CXCL11**  paralleling the recruitment of  **CXCR3**  expressing mononuclear cells.  Ref: 12097412 J Immunol, 2002
- I TAC  [ **CXCL11** ]  induced a marked 4 to 6 fold increase in transendothelial migration TEM of T cells across unstimulated HUVEC from 5.6 to 28% of input T cells and was substantially more active than IFN gamma inducible protein 10, another  **CXCR3**  ligand.  Ref: 12055261 J Immunol, 2002

**Non-interactive relationship** (e.g. studied together, co-existance, homology, etc.)

- RESULTS Expression of IP 10  [ **CXCR3** ] , Mig, I TAC  [ **CXCL11** ] , RANTES,  **CXCR3** , and CCR5, but not MCP 1 and IL 8, increased significantly in both grade 2 and grade 3 rejection P< ; =0.0096.  Ref: 12544874 Transplantation, 2003
- Gastric epithelial cells constitutively expressed mRNA for IP 10  [ **CXCR3** ] , Mig and I TAC  [ **CXCL11** ] .  Ref: 11737065 Clin Exp Immunol, 2001
- Chemokines are produced in a coordinated and sequential manner, with IL 8 and RANTES induced by TNF alpha during early stages, and MCP 1, IP 10  [ **CXCR3** ] , Mig, I TAC  [ **CXCL11** ] , I 309 and MDC induced by IFN gamma during later stages.  Ref: 11876523 Arch Dermatol Res, 2002
- However, because most memory T lymphocytes are also  **CXCR3** , the ability of I TAC  [ **CXCL11** ]  to promote the migration of normal human blood T cells across HUVEC monolayers in Transwell chambers was examined.  Ref: 12055261 J Immunol, 2002
- Induction of the third  **CXCR3**  ligand,  **CXCL11**  I TAC  [ **CXCL11** ]  was not seen in the draining LN.  Ref: 12115662 Eur J Immunol, 2002
- Potential molecular differences between early and late rejection grade > =2 episodes were examined by testing chemokine and receptor gene expression.METHODS Expression of inducible protein IP 10  [ **CXCR3** ] , monokine induced by IFN gamma Mig, interferon inducible T cell alpha chemoattractant  [ **CXCL11** ]  I TAC  [ **CXCL11** ] , regulated on activation normal T cell expressed and secreted RANTES, and their receptors  **CXCR3**  and CCR5 was tested in 60 endomyocardial biopsies from 24 patients using quantitative Taqman real time polymerase chain reaction PCR.  Ref: 12829909 Transplantation, 2003
- The chemokines CXCL9 Mig, CXCL10 IP 10  [ **CXCR3** ] , and  **CXCL11**  I TAC  [ **CXCL11** ]  regulate lymphocyte chemotaxis, mediate vascular pericyte proliferation, and act as angiostatic agents, thus inhibiting tumor growth.  Ref: 12782716 J Exp Med, 2003
- Moreover, cytokines induced IL 15, IP 10  [ **CXCR3** ] , Mig, I TAC  [ **CXCL11** ]  and MIP 3alpha protein accumulation in culture medium from human islets.  Ref: 12627325 Diabetologia, 2003
- CCR3 functional responses are regulated by both  **CXCR3**  and its ligands CXCL9, CXCL10 and  **CXCL11** .  Ref: 12884299 Eur J Immunol, 2003
- Signaling of ORF74 to these proliferative and antiapoptotic signaling pathways can be further modulated positively by growth related oncogene GROalpha CXCL1  [ **CXCL11** ]  and negatively by human gamma interferon inducible protein 10 IP 10  [ **CXCR3** ]  CXCL10, thus acting as an agonist and an inverse agonist, respectively.  Ref: 11799169 J Virol, 2002
- **CXCR3**  internalization following T cell endothelial cell contact preferential role of IFN inducible T cell alpha chemoattractant  **CXCL11** .  Ref: 11739530 J Immunol, 2001
- Lysophosphatidylcholine inhibits T cell specific CXC chemokines IP 10  [ **CXCR3** ] , MIG, and I TAC  [ **CXCL11** ]  expression induced by IFN gamma in human endothelial cells.  Ref: 11795279 Ann N Y Acad Sci, 2001
- CONCLUSIONS Elevated peripheral blood levels of the  **CXCR3**  chemokine ITAC  **CXCL11**  are associated with severe TCAD and may serve as a marker for patients at increased risk for the development of this disease.  Ref: 12695288 Circulation, 2003
- Lesional keratinocytes also overexpressed the ligands of  **CXCR3** , namely, MIG CXCL9, CXCL10, and I TAC  [ **CXCL11** ]   **CXCL11** .  Ref: 12819030 Am J Pathol, 2003
- Interferon gamma inducible chemokines CXCL9 Mig, CXCL10 IP 10  [ **CXCR3** ] ,  **CXCL11**  I TAC  [ **CXCL11** ] , strongly associated to Th1 mediated immune responses, belong to the CXC sub family.  Ref: 12739748 J Endocrinol Invest, 2003
- Remarkably, primary cultures of human microvascular endothelial cells, whose growth is inhibited by CXCL9, CXCL10,  **CXCL11** , and CXCL4, expressed  **CXCR3**  B, but not  **CXCR3**  A.  Ref: 12782716 J Exp Med, 2003
- Differential expression of  **CXCR3**  targeting chemokines CXCL10, CXCL9, and  **CXCL11**  in different types of skin inflammation.  Ref: 11523046 J Pathol, 2001
- However, consistent with its reduced but still considerable ability to down regulate  **CXCR3** , truncated  **CXCL11**  desensitized T cell chemotaxis in response to the intact chemokine.  Ref: 12101279 J Leukoc Biol, 2002
- Immunohistochemical localization confirmed the presence of  **CXCR3**  mononuclear cells within lesions and the presence of the ligand, ITAC  **CXCL11** , on the surface of endothelial cells associated with TCAD.  Ref: 12695288 Circulation, 2003
- I TAC  [ **CXCL11** ]  like IP10  [ **CXCR3** ]  analogs are obtained by substituting the NH 2 terminus residues 1 8 or N loop region residues 12 17 of IP10  [ **CXCR3** ]  with those of I TAC  [ **CXCL11** ] , suggesting that the differences in function of the  **CXCR3**  ligands can be assigned to distinct regions.  Ref: 12417585 J Biol Chem, 2003
- Moreover, in the kidneys of subjects suffering from chronic allograft nephropathy, IP 10  [ **CXCR3** ]  CXCL10, Mig CXCL9 and I TAC  [ **CXCL11** ]   **CXCL11**  have been found to be produced by and to act on the proxymal tubular epithelial cells, endothelial cells and smooth muscle vessel cells, suggesting their possible role in both the genesis of tubular atrophy and allograft artheriosclerosis.  Ref: 12508169 G Ital Nefrol< MedlineTA>G Ital Nefrol, 2003
- Expression and modulation of IFN gamma inducible chemokines IP 10  [ **CXCR3** ] , Mig, and I TAC  [ **CXCL11** ]  in human brain endothelium and astrocytes possible relevance for the immune invasion of the central nervous system and the pathogenesis of multiple sclerosis.  Ref: 12162873 J Interferon Cytokine Res, 2002
- Lyso PC inhibited IFN gamma or IFN gamma plus TNF alpha induced IP 10  [ **CXCR3** ] , Mig, and I TAC  [ **CXCL11** ]  mRNA expression but not that of the IRF 1 or IRF 1 dependent molecule, GBP.  Ref: 11795279 Ann N Y Acad Sci, 2001
- This review discusses chemokine responses in relation to HSV infection of the cornea emphasizing the role of  **CXCR3**  chemokine signaling by the IFN gamma inducible ligands MIG, IP10  [ **CXCR3** ]  and I TAC  [ **CXCL11** ]  and includes discussion of their potential role in immunopathology in the nervous system.  Ref: 12815542 Curr Eye Res, 2003
- Real time, quantitative reverse transcriptase polymerase chain reaction analysis demonstrates that the  **CXCR3**  ligands, interferon IFN inducible protein 10 CXCL10 and IFN inducible T cell alpha chemoattractant  **CXCL11** , are among the many chemokines induced in the adjuvant treated peritoneum.  Ref: 12773510 J Leukoc Biol, 2003
- In this study, we first demonstrate that in COS 7 cells heterologously expressing  **CXCR3** ,  **CXCL11**  is a potent activator of the pertussis toxin sensitive PTX p44 p42 MAPK and Akt PI 3 kinase pathways.  Ref: 12750173 Blood< MedlineTA>Blood, 2003
- As PHA IL 2 treated T cells also express the  **CXCL11**  receptor  **CXCR3** , we investigated whether truncation of  **CXCL11**  would modulate its biological activity for these cells.  Ref: 12101279 J Leukoc Biol, 2002
- Our results demonstrate that among the chemokine mRNAs studied, gamma interferon IFN gamma inducible protein 10 IP 10  [ **CXCR3** ] , monokine induced by IFN gamma MIG, i TAC  [ **CXCL11** ] , lymphotactin, macrophage inflammatory protein 1 beta MIP 1 beta, and RANTES mRNAs were strongly up regulated in infected neonate mice, which correlated with the immunofluorescence staining results showing T cell and macrophage infiltration in the mucosa.  Ref: 11895975 Infect Immun, 2002
- CONCLUSION The expression of IP 10  [ **CXCR3** ] , Mig, I TAC  [ **CXCL11** ] , RANTES,  **CXCR3** , and CCR5 in cardiac allografts significantly correlates with the presence and grade of acute rejection.  Ref: 12544874 Transplantation, 2003
- The CXC chemokine or small inducible cytokine B SCYB subfamily includes the T cell chemoattractants MIG CXCL9, SCYB9, IP 10  [ **CXCR3** ]  CXCL10, SCYB10, and I TAC  [ **CXCL11** ]   **CXCL11** , SCYB11  [ **CXCL11** ] .  Ref: 11685476 Immunogenetics, 2001
- **CXCL11** , we found that even though I TAC  [ **CXCL11** ]  was secreted from IFN gamma activated HSVEC to lower levels than IFN induced protein 10 or the monokine induced by IFN gamma, it was the principal chemokine responsible for  **CXCR3**  internalization.  Ref: 11739530 J Immunol, 2001
- Immunohistochemical localization of the  **CXCR3**  chemokine ligand ITAC  **CXCL11**  on the endothelial surface of TCAD lesions with underlying infiltration of inflammatory mononuclear cells expressing  **CXCR3**  suggests a causative role for this chemokine in the development of TCAD.  Ref: 12695288 Circulation, 2003
- In the absence of IFN gamma, the chemokine response was altered for IP 10  [ **CXCR3** ] , MIG, i TAC  [ **CXCL11** ] , RANTES, and MIP 1 beta mRNAs, while the three ELR C X C chemokine mRNAs studied lipopolysaccharide induced C X C chemokine, MIP 2 alpha, and KC mRNAs were strongly overexpressed.  Ref: 11895975 Infect Immun, 2002
- Moreover, IP 10  [ **CXCR3** ]  and I TAC  [ **CXCL11** ]  cleaved by CD26 DPP IV acted as chemotaxis antagonists and CD26 DPP IV truncated IP 10  [ **CXCR3** ]  and Mig retained their ability to inhibit the angiogenic activity of interleukin 8 in the rabbit cornea micropocket model.  Ref: 11739156 Blood, 2001
- Allografts, but not normal hearts, contained IP 10  [ **CXCR3** ] , Mig, and I TAC  [ **CXCL11** ]  mRNA.  Ref: 12133984 J Immunol, 2002
- The expression of interferon IFN gamma inducible protein IP 10  [ **CXCR3** ] , monokine induced by IFN gamma Mig, interferon inducible T cell alpha chemoattractant  [ **CXCL11** ]  I TAC  [ **CXCL11** ] , regulated on activation normal T cell expressed and secreted RANTES, monocyte chemotactic protein MCP 1, interleukin IL 8, and the receptors  **CXCR3**  and CCR5 were tested using quantitative, real time polymerase chain reaction.  Ref: 12544874 Transplantation, 2003
- Structure activity studies with Mig showed that the extended basic COOH terminal region, which is not present in I TAC  [ **CXCL11** ]  and IP10  [ **CXCR3** ] , is important for binding and activity.  Ref: 12417585 J Biol Chem, 2003
- These results suggest that Lyso PC selectively inhibits IP 10  [ **CXCR3** ] , Mig, and I TAC  [ **CXCL11** ]  expression through IRF 1 independent mechanisms.  Ref: 11795279 Ann N Y Acad Sci, 2001
- As  **CXCR3**  activating chemokines CXCL10 IP 10  [ **CXCR3** ] , CXCL9 Mig, and  **CXCL11**  IP 9  [ **CXCL11** ]  I TAC  [ **CXCL11** ]  specifically attract activated T cells, this study addressed the question of whether differences in the expression of these chemokines correlate with the site and cellular composition of the skin infiltrates in different types of inflammatory skin disease.  Ref: 11523046 J Pathol, 2001
- Persistent elevation of IP 10  [ **CXCR3** ]  and I TAC  [ **CXCL11** ]  was associated with CAV.  Ref: 12133984 J Immunol, 2002
- ... the IFN gamma inducible 10 kDa protein IP 10  [ **CXCR3** ]  and IFN inducible T cell alpha chemoattractant I TAC  [ **CXCL11** ]  are induced only after inflammatory stimuli.  Ref: 12162873 J Interferon Cytokine Res, 2002
- Therefore, we investigated expression of IFN inducible protein 10 IP 10  [ **CXCR3** ] , IFN inducible T cell alpha chemoattractant I TAC  [ **CXCL11** ] , monokine induced by IFN Mig, and their receptor  **CXCR3**  in consecutive endomyocardial biopsies n = 133 from human cardiac allografts and corresponding normal donor hearts n = 11 before transplantation.  Ref: 12133984 J Immunol, 2002
- Intragraft vascular cells expressed high levels of IP 10  [ **CXCR3** ]  and I TAC  [ **CXCL11** ] , while Mig localized predominantly in infiltrating macrophages.  Ref: 12133984 J Immunol, 2002
- We found that elevated levels of MIG CXCL9, IFN inducible protein 10 IP 10  [ **CXCR3** ]  CXCL10, and ITAC  **CXCL11**  in human bronchoalveolar lavage fluid were associated with the continuum from acute to chronic rejection.  Ref: 12097412 J Immunol, 2002
